# Parental and cell-division origin analysis to reduce false-positives in mosaic embryos for preimplantation genetic testing

**DOI:** 10.1093/hropen/hoaf075

**Published:** 2025-12-01

**Authors:** Qi Zhang, Guicen Liu, Yezhou Xiang, Yangyun Zou, Yulin Chen, Yingying Xia, Shun Xiong, Tao Fu, Jiang Wang, Yan Jiang, Jiaojiao Xiong, Xiaodong Zhang, Sijia Lu, Dongyun Liu, Guoning Huang, Tingting Lin

**Affiliations:** Chongqing Key Laboratory of Human Embryo Engineering and Precision Medicine, Center for Reproductive Medicine, Women and Children’s Hospital of Chongqing Medical University, Chongqing, China; Chongqing Clinical Research Center for Reproductive Medicine, Chongqing Health Center for Women and Children, Chongqing, China; Chongqing Key Laboratory of Human Embryo Engineering and Precision Medicine, Center for Reproductive Medicine, Women and Children’s Hospital of Chongqing Medical University, Chongqing, China; Chongqing Clinical Research Center for Reproductive Medicine, Chongqing Health Center for Women and Children, Chongqing, China; Chongqing Key Laboratory of Human Embryo Engineering and Precision Medicine, Center for Reproductive Medicine, Women and Children’s Hospital of Chongqing Medical University, Chongqing, China; Chongqing Clinical Research Center for Reproductive Medicine, Chongqing Health Center for Women and Children, Chongqing, China; Yikon Genomics Co., Ltd., Suzhou, China; Yikon Genomics Co., Ltd., Suzhou, China; Yikon Genomics Co., Ltd., Suzhou, China; Chongqing Key Laboratory of Human Embryo Engineering and Precision Medicine, Center for Reproductive Medicine, Women and Children’s Hospital of Chongqing Medical University, Chongqing, China; Chongqing Clinical Research Center for Reproductive Medicine, Chongqing Health Center for Women and Children, Chongqing, China; Chongqing Key Laboratory of Human Embryo Engineering and Precision Medicine, Center for Reproductive Medicine, Women and Children’s Hospital of Chongqing Medical University, Chongqing, China; Chongqing Clinical Research Center for Reproductive Medicine, Chongqing Health Center for Women and Children, Chongqing, China; Chongqing Key Laboratory of Human Embryo Engineering and Precision Medicine, Center for Reproductive Medicine, Women and Children’s Hospital of Chongqing Medical University, Chongqing, China; Chongqing Clinical Research Center for Reproductive Medicine, Chongqing Health Center for Women and Children, Chongqing, China; Center for Prenatal Diagnosis, Chongqing Health Center for Women and Children, Women and Children’s Hospital of Chongqing Medical University, Chongqing, China; Center for Prenatal Diagnosis, Chongqing Health Center for Women and Children, Women and Children’s Hospital of Chongqing Medical University, Chongqing, China; Chongqing Key Laboratory of Human Embryo Engineering and Precision Medicine, Center for Reproductive Medicine, Women and Children’s Hospital of Chongqing Medical University, Chongqing, China; Chongqing Clinical Research Center for Reproductive Medicine, Chongqing Health Center for Women and Children, Chongqing, China; Yikon Genomics Co., Ltd., Suzhou, China; Chongqing Key Laboratory of Human Embryo Engineering and Precision Medicine, Center for Reproductive Medicine, Women and Children’s Hospital of Chongqing Medical University, Chongqing, China; Chongqing Clinical Research Center for Reproductive Medicine, Chongqing Health Center for Women and Children, Chongqing, China; Chongqing Key Laboratory of Human Embryo Engineering and Precision Medicine, Center for Reproductive Medicine, Women and Children’s Hospital of Chongqing Medical University, Chongqing, China; Chongqing Clinical Research Center for Reproductive Medicine, Chongqing Health Center for Women and Children, Chongqing, China; Chongqing Key Laboratory of Human Embryo Engineering and Precision Medicine, Center for Reproductive Medicine, Women and Children’s Hospital of Chongqing Medical University, Chongqing, China; Chongqing Clinical Research Center for Reproductive Medicine, Chongqing Health Center for Women and Children, Chongqing, China

**Keywords:** mosaicism, uneven score, parental origin, cell-division origin, false-positives

## Abstract

**STUDY QUESTION:**

Can parental and cell-division origin analysis identify the false-positive chromosomal aberrations in mosaic embryos detected during preimplantation genetic testing for aneuploidy (PGT-A)?

**SUMMARY ANSWER:**

Parental origin analysis reclassified over half of mosaic embryos as euploid to reduce diagnostic uncertainty, and cell-division origin analysis effectively differentiated mitotic from meiotic errors to guide evidence-based mosaic embryo transfer.

**WHAT IS KNOWN ALREADY:**

Mosaic embryos pose significant challenges in PGT-A, with reported detection rates varying widely (2–35.6%) due to biological and technical factors, but current methods lack reliable approaches to distinguish true mosaicism from technical artifacts. Decisions regarding mosaic embryo transfer remain conservative and hampered by inadequate understanding of the origins of errors (mitotic vs meiotic) and limited data on long-term neonatal outcomes.

**STUDY DESIGN, SIZE, DURATION:**

In this retrospective study, we analyzed 9062 PGT-A results and 8645 amniocentesis samples from 2021 to 2024 to investigate the difference in mosaicism rates between PGT treatment and prenatal diagnosis. An analysis of parental and cell-division origins was performed on 1221 consecutive results from PGT-A and PGT for monogenic disorders (PGT-M) from 259 patients across 304 treatment cycles in 2024. Multi-site re-biopsies of 36 donated embryos and the clinical outcomes of 19 mosaic embryo transfers were analyzed in 2024 and 2025.

**PARTICIPANTS/MATERIALS, SETTING, METHODS:**

An innovative algorithm, termed parental haplotype trace (PH-trace), was developed to identify the genetic origin of chromosomal aberrations for mosaic verification. Briefly, biallelic homozygous single-nucleotide polymorphisms (SNPs) in the parental genome exhibit equal allelic frequencies in euploid embryos. When chromosomal aberrations occur, these SNPs show an allelic bias toward either the maternal or paternal genome. We defined the ratio of maternal-biased SNPs to paternal-biased SNPs as the uneven score to quantitatively assess the parental origin of chromosomal aberrations. Receiver operating characteristic (ROC) curve analysis based on uneven scores from euploid and aneuploid embryos was used to determine critical thresholds for identifying false-positive mosaic embryos. Additionally, heterozygous SNPs in the parental genome were used to determine the cell-division origin of chromosomal aberrations. To validate our findings, we performed multi-site re-biopsies of aneuploid and mosaic embryos.

**MAIN RESULTS AND THE ROLE OF CHANCE:**

The prevalence of mosaicism differed significantly between PGT-A and prenatal diagnosis (12.2% vs 0.9%, *P *< 0.001). Parental origin analysis based on PH-trace reclassified 52.6% of mosaic embryos as euploid and increased the overall rate of usable embryos by 8.6%. Mitotic errors accounted for the majority of true mosaic cases, providing critical guidance for embryo transfer prioritization. Re-biopsy validation of donated embryos revealed that 94.1% of predicted false-positive copy-number variations (CNVs) were not detected in subsequent samples, whereas 71.4% of parental-biased CNVs were repeatedly detected. Among mosaic embryos resulting in live births, 66.7% were false-positive, and 22.2% originated from mitotic errors.

**LIMITATIONS, REASONS FOR CAUTION:**

Our two-tiered analytical approach relies on a sufficient number of informative SNPs in parental and embryonic genomes (e.g. over 30 informative SNPs per chromosomal aberration). Additionally, the clinical implications of this method require further validation through long-term follow-up studies.

**WIDER IMPLICATIONS OF THE FINDINGS:**

This study presents an effective strategy to identify false-positive mosaic embryos, thereby improving embryo utilization in clinical treatment. By elucidating the cell-division origin of mosaicism, our findings provide embryologists with evidence-based criteria for prioritizing embryo transfers. Furthermore, this approach may reduce the number of IVF cycles and associated costs for patients with limited euploid embryo availability.

**STUDY FUNDING/COMPETING INTEREST(S):**

This work was supported by the National Natural Science Foundation of China (82371728), the Chongqing’s Mid-young Medical Elite Talent Project (YXGD202555), the Key Projects of the Collaborative Medical Research between Science and Health Commission of Chongqing (2026ZDXM004), the Chongqing Municipal Technological Innovation and Application Development Special Project (CSTB2022TIAD-KPX0146), and the Chongqing Nature Science Foundation (CSTB2023NSCQ-MSX0443). The authors have no competing interests to declare.

**TRIAL REGISTRATION NUMBER:**

N/A

WHAT DOES THIS MEAN FOR PATIENTS? Preimplantation genetic testing (PGT) of embryos generated by IVF sometimes finds embryos to be ‘mosaic’, meaning they have a mix of cells with normal and abnormal chromosomes. This can create uncertainty about whether the embryo is suitable for transfer.

Our study introduces a new method to clarify this uncertainty. By tracing the genetic material from each parent, we found that more than half of the embryos originally labeled as mosaic were actually normal. This can significantly increase the number of embryos available for transfer after IVF.

For embryos that are truly mosaic, our method can also identify the likely cause. Most of these cases are due to errors that occur after fertilization, which are often associated with better outcomes than errors that occur before fertilization. This provides doctors with valuable evidence to help prioritize which mosaic embryos have the best potential for a successful pregnancy.

In summary, this new approach helps to reduce diagnostic uncertainty, increases the number of usable embryos, and offers clearer guidance for embryo selection, potentially making the PGT journey more efficient and hopeful for patients.

## Introduction

Chromosomal aberrations in preimplantation embryos constitute a principal etiological factor in human reproductive inefficiency, manifesting as implantation failure, miscarriages, and congenital disorders (Greaney *et al.*, 2018; Gruhn *et al.*, 2019). Preimplantation genetic testing (PGT) has transformed ART outcomes through comprehensive screening for chromosomal aneuploidies (PGT-A), monogenic disorders (PGT-M), and chromosomal structural rearrangements (PGT-SR) (Zegers-Hochschild *et al.*, 2017; Cornelisse *et al.*, 2020; Yan *et al.*, 2021). However, mosaic embryos, characterized by the presence of two or more chromosomally distinct cell populations, remain widely controversial, involving mosaicism reporting guidelines, inter-laboratory variability in detection rates, technical artifacts, and clinical management (Popovic *et al.*, 2020; Treff and Marin, 2021; Leigh *et al.*, 2022).

At present, the prevailing mosaic classification paradigm relies on intermediate copy-number thresholds derived from next-generation sequencing (Treff and Marin, 2021; Campos, 2023). Embryos are typically classified according to the proportion of aneuploid cells: those with proportions exceeding 70% or 80% are defined as aneuploid; those with less than 20% or 30% are considered euploid; and those falling within the intermediate range (20–80% or 30–70%) are classified as mosaic. Due to the biological, technical, analytical, and clinical complexities involved in interpreting PGT-A results, inter-laboratory detection rates of mosaic embryos vary substantially, ranging from 2% to 35.6% (Nakhuda *et al.*, 2018; Rana *et al.*, 2023; Popovic *et al.*, 2024). This wide variability has raised concerns about technical artifacts and the overall accuracy of PGT-A (Gleicher *et al.*, 2021; Wu *et al.*, 2021). True biological mosaicism mainly originates from post-zygotic mitotic errors or the rescue of meiotic errors (Ottolini *et al.*, 2015; Levy *et al.*, 2021). Previous studies have estimated the incidence of true mosaicism to be less than 1% in human preimplantation blastocysts, less than 2% in prenatal samples, and less than 0.2% in live births (Huang *et al.*, 2009; Malvestiti *et al.*, 2015; Zamani Esteki *et al.*, 2019). These diagnostic discrepancies highlight the urgent need to identify false-positive cases of mosaicism and reconsider the reporting guidelines for mosaicism in PGT-A.

Since 2016, clinical practice has gradually adopted mosaic embryo transfer (Greco *et al.*, 2015), which has been further supported by the comparable outcomes to euploid transfer when morphological grading is taken into account (Munné *et al.*, 2019; Capalbo *et al.*, 2021; Viotti *et al.*, 2023). Our previous retrospective analysis of 2850 PGT-A results revealed significant correlations between trophectoderm (TE) grade, PGT laboratory protocols, and mosaicism detection rates (Zhang *et al.*, 2024b), and the transfer of 98 mosaic embryos resulted in 51.0% and 39.4% clinical pregnancy and live birth rates, respectively. However, the current selection criteria for mosaic transfer remain empirically derived, incorporating mosaic type (whole, segmental or complex), aneuploid ratio, and limited clinical outcome data (Viotti *et al.*, 2021; Cheng *et al.*, 2022; De Rycke *et al.*, 2022; Practice Committees of the American Society for Reproductive Medicine and the Genetic Counseling Professional Group, 2023). Many reproductive clinics are unwilling to transfer mosaic embryos due to safety concerns (Muñoz *et al.*, 2024). This conservative practice reflects three key evidence gaps: (1) the absence of standardized methods to distinguish technical artifacts from true mosaicism, (2) inadequate understanding of the origins of chromosomal errors (mitotic vs meiotic), and (3) limited data on long-term neonatal outcomes (Treff and Franasiak, 2017; Eggenhuizen *et al.*, 2021; Waldvogel *et al.*, 2024).

In this study, we retrospectively analyzed 9062 PGT-A results from February 2021 to August 2024 and 8645 amniocentesis samples from October 2022 to October 2024 to reveal the significant difference in mosaicism prevalence between PGT-A and prenatal diagnosis (12.2% vs 0.9%, *P *< 0.001). We hypothesize that parental and cell-division origin analysis of chromosomal aberrations can effectively distinguish false-positive mosaic embryos and guide their transfer. Briefly, biallelic homozygous single-nucleotide polymorphisms (SNPs) in the parental genomes exhibit balanced allelic frequencies in euploid embryos, whereas a parental-allelic bias occurs in true chromosomal aberrations. We defined the ratio of maternal-biased SNPs to paternal-biased SNPs as an uneven score to quantitatively assess this bias. Based on the data from 767 euploid and 187 aneuploid embryos, an uneven score curve was plotted, and the critical values were determined through receiver operating characteristic (ROC) curve to evaluate the parental origin of chromosomal aberrations. We then applied this algorithm to reanalyze 94 aneuploid-mosaic and 173 mosaic embryos, and redefined 52.6% of the mosaic embryos as euploid, reducing the mosaic rate from 14.2% to 6.4% and increasing the proportion of usable embryos by 8.6%. Cell-division origin analysis was performed based on heterozygous SNPs in the parental genome and revealed that mosaic chromosomal aberrations mainly originate from mitotic errors. The revalidation of donor embryos also confirmed the results, and clinical outcomes of mosaic embryo transfer provided supporting evidence. Thus, we defined this innovative algorithm as parental haplotype trace (PH-trace) to identify the parental and cell-division origin of chromosomal aberrations for mosaic verification. Our study provides a feasible approach to distinguish technical artifacts from true mosaicism to reduce diagnostic uncertainty, and guides evidence-based decisions of mosaic embryo transfer by differentiating meiotic from mitotic errors.

## Materials and methods

### Study participants and ethics statement

**Figure 1.**
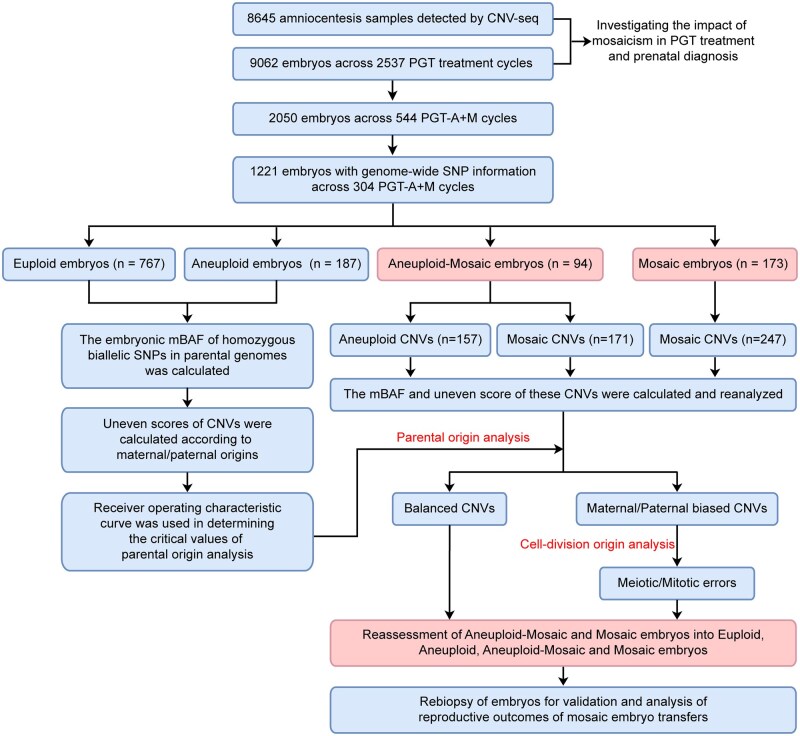
**Flow chart of this study.** First, we recruited 2537 PGT treatment cycles with 9062 embryos and 8645 amniocentesis samples to investigate the impact of mosaicism in PGT treatment and prenatal diagnosis. Next, we focused on 304 cycles of PGT-A combined with PGT-M to get whole-genome SNPs information for parental origin analysis, that euploid and aneuploid embryos were referred for the uneven score curve drawing and decision of critical values to assess aneuploid-mosaic and mosaic embryos. After that, the parental and cell-division origin was analyzed in aneuploid and mosaic CNVs. Finally, re-biopsies of aneuploid and mosaic embryos were conducted to validate the results, and the reproductive outcomes of mosaic embryo transfers were summarized. CNVs, copy-number variations; PGT-A, preimplantation genetic testing for aneuploidy; PGT-M, preimplantation genetic testing for monogenic disorders; SNPs, single-nucleotide polymorphisms.

In this study, we reviewed 2537 PGT-A treatment cycles, including 1220 PGT-A only cycles, 773 PGT-A + SR cycles, and 544 PGT-A + M cycles, conducted at the Center for Reproductive Medicine, Women and Children’s Hospital of Chongqing Medical University (Chongqing, China) from February 2021 to August 2024. We also retrospectively analyzed 8645 amniocentesis samples detected by copy-number variation sequencing (CNV-seq) at the Center for Prenatal Diagnosis, Women and Children’s Hospital of Chongqing Medical University from October 2022 to October 2024. The study design is illustrated in Fig. 1. For parental origin analysis, only the PGT-A + M cycles with genome-wide SNP information were included, while the PGT-A + M cycles with insufficient SNP information (<30) were excluded. For ROC curve analysis, the autosomes from euploid embryos were used as the balanced reference group, whereas the whole and segmental chromosomal CNVs from aneuploid embryos, along with the X chromosome of male euploid (46, XY) embryos, were considered as the trisomy and monosomy group. CNV results with poor sequencing quality and less than 30 informative SNPs were excluded. Ethical approval for this study was obtained from the Clinical Application and Ethics Committee of Human Assisted Reproductive Technology at Chongqing Health Center for Women and Children (approval number: 2024-RGI-02; date of approval: 17 October 2024). We have also obtained written informed consent from the embryo donors.

### CNV and haplotype analysis in PGT treatment

Genomic DNA (gDNA) was extracted from peripheral blood samples of couples and their family members as previously reported (Zhang *et al.*, 2024a). The expanded blastocyst was held with a pipette (Sunlight Medical Inc., Jacksonville, FL, USA), and the zona pellucida (ZP) was punctured by a laser pulse (Zilos-tk, Hamilton Thorne Ltd., Beverly, MA, USA) to aspirate four to six TE cells. Then, whole-genome amplification (WGA) was performed on both gDNA and TE biopsies using the MALBAC assay kit (Yikon Genomics Co., Ltd., Suzhou, China), and subsequently, the prepared DNA libraries were sequenced on the Illumina HiSeq 2500 platform (Illumina, Inc., San Diego, CA, USA) at a depth of ≥ 2×. For CNV analysis, the clean data were aligned with the reference genome (GRCh37), and a circular binary segmentation algorithm was applied to normalize the read count values (R2) of embryos for comparison with the normalized read count values (R1) in the reference library. Finally, the copy number (CN) of each region in the embryos was calculated using the formula CN = 2 × (R2/R1) and classified into five categories: duplications (CN ≥ 2.7), mosaic duplication (2.7 > CN ≥ 2.3), normal region (2.3 > CN > 1.7), mosaic deletion (1.7 ≥ CN > 1.3) and deletion (CN ≤ 1.3). The reported duplication and deletion exceed 4 Mb, while the mosaic duplication and deletion exceed 10 Mb. For haplotype analysis, SAMtools (v1.16) (Li *et al.*, 2009) and GATK (v4.2.0) (McKenna *et al.*, 2010) were used to extract whole-genome haplotypes. A likelihood-based haplotyping approach was used to determine the most probable haplotype configurations for embryos and other family members.

### Prenatal diagnosis

In mid-pregnancy, amniocentesis was performed at the Center for Prenatal Diagnosis, and gDNA was extracted from amniotic fluid as previously described (Zhang *et al.*, 2024a). Then, a gDNA library was constructed and sequenced on the AmCareSeq-2000 platform (AmCare Genomics, Guangzhou, China). The sequencing strategy involved insertion sizes of 200–500 bp, paired-ends of 150 bp, depths of >5×, and raw reads of >100 Mb per sample. An internal bioinformatics pipeline (AmCare Genomics) was applied to CNVs analysis, and the coverage spectra were generated for 25 kb window across the genome to normalize with a gender-matched cohort (greater than or equal to five males and five females) and calculate *Z*-scores. If four or more consecutive 25 kb windows showed *Z*-scores < –2 or > 2, CNVs were flagged for manual review to achieve a resolution of 100 kb with a 25 kb bin size. The clinical significance of CNVs was assessed according to the American College of Medical Genetics guidelines (Richards *et al.*, 2015).

### Parental and cell-division origin analysis of chromosomal aberrations

Our study applied Mendelian inheritance principles to analyze chromosomal aberrations in embryo-based SNP patterns. In euploid embryos, homozygous biallelic SNPs (e.g. parental AA × BB) consistently yield heterozygous offspring (A:B = 1:1) with B-allele frequency (BAF) approximating 0.50 (Supplementary Fig. S1a). We developed a modified metric, maternal BAF (mBAF), calculated as the maternal allele count divided by the total parental allele count. Chromosomal aberrations disrupt this balance, causing characteristic mBAF deviations, such as monosomies showing mBAF of zero (maternal loss) or one (paternal loss) with loss of heterozygosity (LOH), while trisomies exhibit mBAF of 0.33 (paternal gain) or 0.67 (maternal gain) (Supplementary Fig. S1b).

To precisely determine the cell-division origin, we analyzed informative SNPs where one parent was heterozygous (AB) and the other homozygous (AA/BB) (Supplementary Fig. S2a). The origin of monosomy was distinguished based on heterozygosity patterns: retained heterozygosity indicated mitotic errors, whereas LOH was suggestive of meiotic errors (Supplementary Fig. S2b). In the case of trisomy, the extra allele of mitotic origin will lose heterozygosity because it comes from the same homologous chromosome, a pattern defined as a single parental homolog (SPH) (Supplementary Fig. S2a and c). For the meiotic-origin trisomy, two different homologous chromosomes from the same heterozygous parent are present, a pattern defined as both parental homologs (BPH). This configuration arises due to synapsis and crossover events during meiosis and exhibits a characteristic genomic signature (Supplementary Fig. S2a and c). Furthermore, centromere-containing BPH regions indicate meiosis I errors, while other BPH patterns suggest meiosis II errors (Supplementary Fig. S2c). This systematic approach enables a comprehensive classification of error mechanisms in embryonic chromosomal aberrations.

### Re-biopsy of the blastocyst

To validate our findings, donated aneuploid and mosaic embryos were re-biopsied. For aneuploid embryos originating from meiotic or mitotic errors, TE cells from three different positions and inner cell mass (ICM) cells were sampled. Briefly, the expanded blastocyst was held using a pipette (Sunlight Medical Inc.) with the ICM positioned at 9 o’clock orientation. Then the ZP was punctured by a laser pulse (Zilos-tk, Hamilton Thorne Ltd.) to aspirate four to six TE cells at 3 o’clock, 6 o’clock, and 12 o’clock, respectively (Supplementary Fig. S3). Moreover, approximately 10 ICM cells were collected separately. Finally, the four biopsied samples from each embryo were washed three times in G-Mops plus (Vitrolife, Gothenburg, Sweden) and transferred into PCR tubes for cryopreservation. Additionally, re-biopsies were performed on mosaic embryos and aneuploid embryos exhibiting false-positive chromosomal aberrations to validate the inferred parental origin of the chromosomal aberrations. Similarly, TE cells were biopsied from the 12 o’clock and 3 o’clock positions, and the residual embryo was separately preserved for subsequent analysis. WGA and DNA library preparation of each TE and ICM biopsy, and sequencing data analysis were performed as a conventional PGT treatment.

### Frozen embryo transfer and clinical outcomes

In PGT treatments, a freeze-all strategy was adopted, and hormone replacement therapy was applied to frozen embryo transfer (FET). Briefly, when serum estradiol and progesterone levels were below 50 pg/ml and 1 ng/ml, respectively, hormone therapy was initiated on the second or third days of the menstrual cycle with oral administration of 2–5 mg estradiol valerate (Progynova, Bayer Schering Pharma AG, Leverkusen, Germany) daily for 14 days until endometrial thickness reached 7 mm. Subsequently, 5 days prior to FET, 600 mg of vaginal micronized progesterone (Utrogestan, Besins Healthcare, Monaco) was taken daily for luteal phase support until the 12th week of pregnancy. On the day of FET, 20 mg dydrogesterone (Duphaston, Abbott, Hoofddorp, Netherlands) was orally administered. Clinical pregnancy is defined as the presence of a gestational sac on the 28th day after FET. Early miscarriage refers to a miscarriage that occurs before 12 weeks of pregnancy, and live birth was defined as giving birth to a live newborn.

### Statistical analysis

Statistical analyses were conducted using GraphPad Prism version 8.0.2 (GraphPad Software, San Diego, CA, USA) and R version 4.4.3 (R Foundation for Statistical Computing, Vienna, Austria; https://www.r-project.org). Differences between the two groups were assessed using chi-square tests. All data were presented as frequencies (n) and percentages (%), and *P *< 0.05 was considered statistically significant. ROC analysis was used to determine the critical value for parental chromosomal bias.

## Results

### The prevalence of mosaicism in PGT treatment and prenatal diagnosis

**Figure 2.**
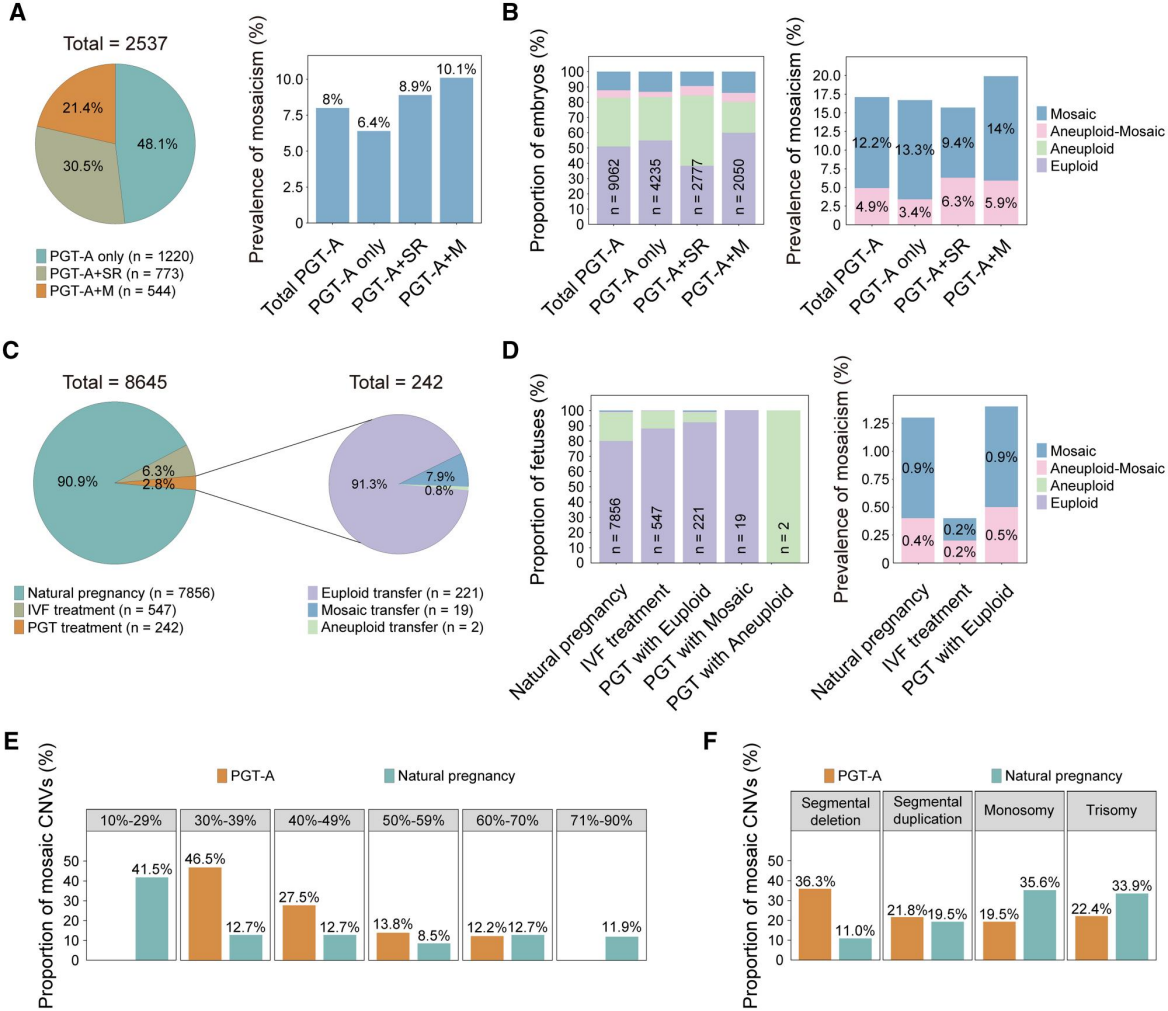
**Prevalence of mosaicism in PGT treatment and prenatal diagnosis.** (**A**) Overview of recruited PGT treatment cycles. PGT-A + SR: PGT-A and PGT-SR were implemented together; PGT-A + M: PGT-A and PGT-M were implemented together. The proportion of mosaicism represents the cycles containing mosaic embryos but no euploid embryos. (**B**) The proportion of different embryos in the recruited PGT cycles, including euploid, aneuploid, aneuploid-mosaic, and mosaic embryos. The prevalence of mosaicism across the embryos was shown. (**C**) The description of recruited prenatal samples in natural pregnancies, IVF, and PGT treatment. The embryos transferred in PGT treatment were divided into euploid, mosaic, and aneuploid embryos, respectively. (**D**) The proportion of euploid, aneuploid, aneuploid-mosaic, and mosaic fetuses in prenatal diagnosis. The prevalence of aneuploid-mosaic and mosaic fetuses in natural pregnancy, IVF, and euploid transfer is shown. No mosaicism was observed in the mosaic and aneuploid transfer groups. (**E**) Distribution of CNVs with different mosaic ratios in PGT-A treatment and prenatal diagnosis of natural pregnancies. (**F**) Distribution of CNVs with different chromosomal types in PGT-A treatment and prenatal diagnosis of natural pregnancies. PGT-A, preimplantation genetic testing for aneuploidy; PGT-M, preimplantation genetic testing for monogenic disorders; PGT-SR, preimplantation genetic testing for chromosomal structural rearrangements; CNVs, copy-number variations.

The flow chart for this retrospective study is shown in Fig. 1. First, we investigated the prevalence of embryonic mosaicism across 2537 PGT-A treatment cycles comprising 1220 PGT-A only cycles, 773 PGT-A + SR cycles, and 544 PGT-A + M cycles (Fig. 2a and Supplementary Table S1). A critical finding was that 8.0% of all cycles, ranging from 6.4% to 10.1% across subgroups, faced the clinical dilemma of having only mosaic embryos available for transfer. Embryo-level analysis of the 9062 biopsied samples revealed that 51.0% were euploid, while 17.1% exhibited mosaicism, including 12.2% which were only mosaic, and 4.9% which were aneuploid-mosaic (Fig. 2b and Supplementary Table S2). The prevalence of only mosaic embryos ranged from 9.4% to 14.0%, while that of aneuploid-mosaic embryos ranged from 3.4% to 6.3% across subgroups. These findings demonstrate that mosaicism affects 15–20% of tested embryos and 6–10% of PGT treatment cycles.

To evaluate the prevalence of mosaicism in prenatal diagnosis, we analyzed 8645 amniocentesis samples detected by CNV-seq, including 7856 natural pregnancies, 547 IVF-derived pregnancies, and 242 PGT-derived pregnancies (Fig. 2c and Supplementary Table S2). Strikingly, all 19 pregnancies from mosaic embryo transfers resulted in euploid fetuses (Fig. 2d and Supplementary Fig. S4a). The two aneuploid embryos that were transferred developed into aneuploid fetuses as expected, carrying parental CNVs of uncertain clinical significance (Fig. 2d and Supplementary Fig. S4b). Surprisingly, the overall mosaicism rate in amniotic fluid was markedly lower in natural pregnancies than that in PGT embryos (0.9% vs 12.2%, *P *< 0.001) (Fig. 2b and d), suggesting the presence of technical artifacts in PGT testing or the occurrence of effective embryonic self-correction. The occurrence of 6.8% aneuploidy after PGT euploid embryo transfer is mainly due to the sensitivity difference between PGT testing and prenatal diagnosis (Supplementary Tables S2 and S3).

More than 70% of the mosaic CNVs had a ratio of <50% mosaicism (Fig. 2e). The PGT samples peaked at a 30–39% ratio of mosaicism, whereas natural pregnancies peaked at a 10–29% ratio (Fig. 2e and Supplementary Table S4). Furthermore, monosomy and trisomy predominated in natural pregnancies, while segmental CNVs were more frequently observed in PGT samples (Fig. 2f and Supplementary Table S4). Natural pregnancy mosaicism exhibited a sex chromosome preference, while PGT mosaicism was genome-wide distributed (Supplementary Fig. S5). Collectively, these findings reveal systematic differences in mosaic characteristics between PGT samples and naturally conceived pregnancies.

### Establishment of the PH-trace strategy based on euploid and aneuploid embryos

**Figure 3.**
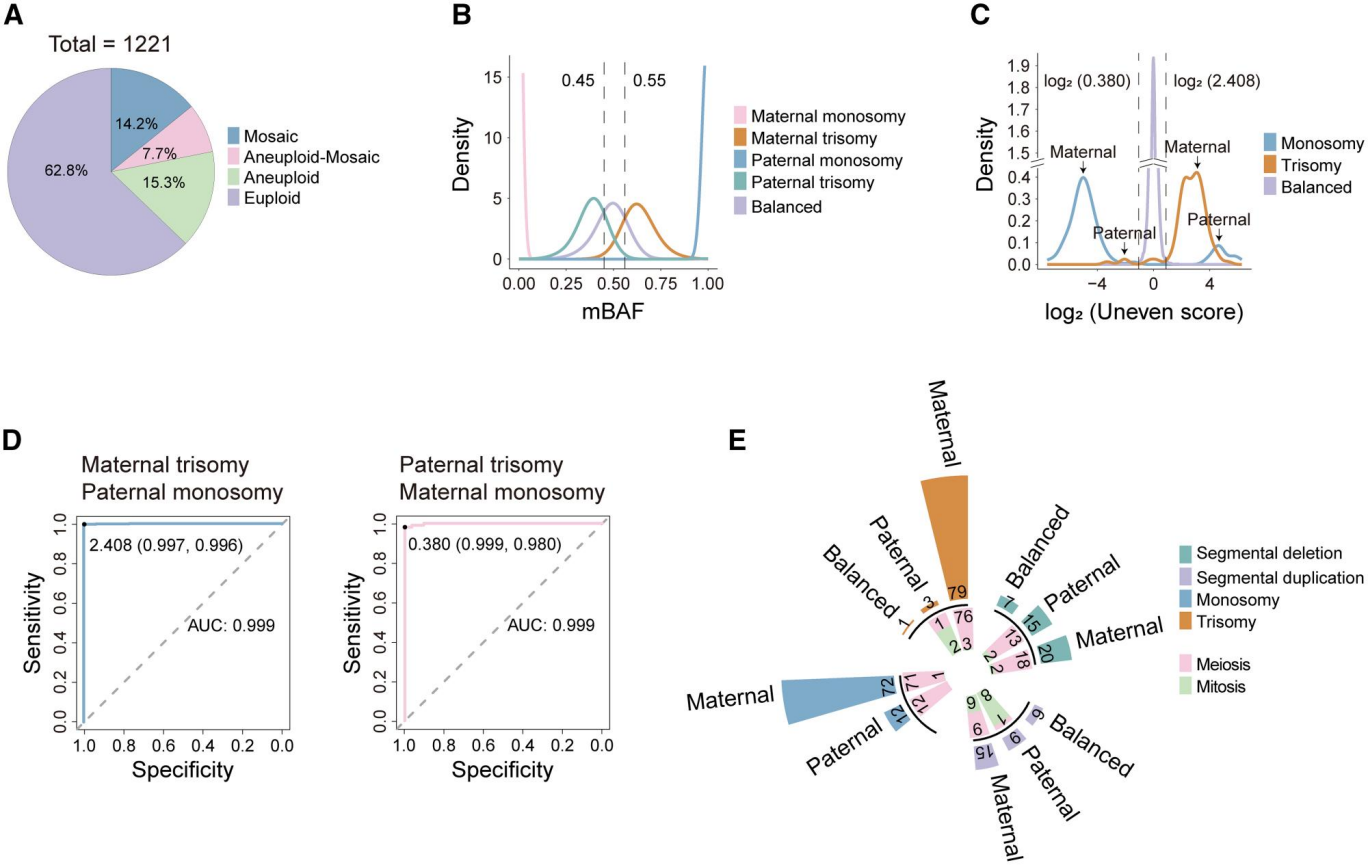
**Distribution curve of mBAF and uneven scores in euploid and aneuploid embryos.** (**A**) The overview of the 1221 embryos with CNV results and whole-genome SNP information. (**B**) The mBAF density map of five chromosome subgroups, balanced (close to 0.50), maternal trisomy (close to 0.67), maternal monosomy (close to zero), paternal trisomy (close to 0.33), and paternal monosomy (close to one), according to euploid and aneuploid embryos. mBAF was derived using the count of maternal allele as the numerator and the sum of maternal and paternal alleles as the denominator. The threshold range of SNPs with balanced parental origin was set as 0.45–0.55 in this study, while others were defined as parental biased. (**C**) The number of maternal-biased SNPs (Nmat) divided by the number of paternal-biased SNPs (Npat) was defined as uneven score (Nmat/Npat), and the distribution curves of balanced, monosomy, and trisomy chromosomes were calculated by log2(uneven score). (**D**) The receiver operating characteristic curves of uneven scores. For maternal trisomy and paternal monosomy, it was analyzed with 7786 euploid chromosomes and 505 aneuploid chromosomes. For paternal trisomy and maternal monosomy, we compared 8378 euploid chromosomes and 102 aneuploid chromosomes. The critical value for maternal trisomy and paternal monosomy was set as >2.408 with an AUC of 0.999, while the critical value for paternal trisomy and maternal monosomy was set as <0.380 with an AUC of 0.999. (**E**) Summary of 187 aneuploid embryos carrying 262 CNVs, including monosomy (n = 84), trisomy (n = 83), segmental deletion (n = 55), and segmental duplication (n = 40). CNVs, copy-number variations; BAF, B-allele frequency; mBAF, maternal BAF; SNP, single-nucleotide polymorphism.

According to the analytical strategies outlined in Supplementary Figs S1 and S2, we then developed a comprehensive SNP-based approach to investigate the parental origin of chromosomal aberrations in 1221 embryos from 259 patients across 304 consecutive PGT-A + M cycles (Fig. 1). The cohort included 767 euploid, 187 aneuploid, 94 aneuploid-mosaic, and 173 mosaic embryos (Figs 1 and 3a). First, we assessed the mBAF of SNPs that were homozygous with biallelic alleles in parental genomes to analyze their patterns in euploid and aneuploid embryos. The density distribution of mBAF values divided these SNPs into five subgroups: balanced, maternal trisomy, maternal monosomy, paternal trisomy, and paternal monosomy (Fig. 3b). We then defined an mBAF threshold range of 0.45–0.55 to identify balanced SNPs, with values falling outside this range indicating parental bias.

To quantify parental origin bias of chromosomal aberrations, we used the ratio of maternal-biased to paternal-biased SNPs (Nmat/Npat) as an ‘uneven score’ metric for this region. The analysis of uneven score distribution across euploid and aneuploid chromosomes revealed a clear separation among the balanced, monosomic and trisomic subgroups (Fig. 3c). Then, ROC curve analysis identified highly discriminative critical values for maternal trisomy and paternal monosomy as >2.408 (AUC = 0.999), and for paternal trisomy and maternal monosomy as <0.380 (AUC = 0.999) (Fig. 3d). Chromosomal aberrations with uneven scores between 0.380 and 2.408 are classified as balanced inheritance, indicating false-positive possibility. During this process, we identified three exceptional cases among euploid embryos: one suggesting maternal genome-wide uniparental disomy (UPD; mBAF ≈ 1), one indicating maternal triploidy (mBAF ≈ 0.67), and one exhibiting single-chromosome UPD (Supplementary Fig. S6a). Additionally, the two aneuploid embryos carrying three false-positive CNVs were re-identified as euploid (Table 1 and Supplementary Fig. S6b). We defined this innovative algorithm as PH-trace to identify the parental origin of chromosomal aberrations based on critical values of uneven scores.

**Table 1.** The results of trophectoderm cell biopsies and testing of the remaining whole embryos.

ID	Clinical TE biopsy	Parental origin	Cell-division origin	**TE**_**1**	**TE**_**2**	Remaining embryo testing
1	dup(16)(q24.1q24.3)(∼4.20 Mb)	Balanced	NA	Euploid	Euploid	Euploid
2	+11	Balanced	NA	Euploid	Euploid	Euploid
	del(22)(q11.1q12.3)(∼21Mb)	Balanced	NA	Euploid	Euploid	Euploid
3	del(1)(q32.1q44)(∼50.45 Mb,∼33%)	Balanced	NA	Euploid	Euploid	Euploid
	+20(∼65%)	Maternal	Mitosis			
	dup(X)(p21.1)(∼1.20 Mb)	Maternal	Mitosis			
4	dup(10)(p15.3p11.21)(∼37.20 Mb,∼30%)	Balanced	NA	Euploid	Euploid	Euploid
5	del(5)(q22.3q35.1)(∼58Mb,∼30%)	Balanced	NA	Euploid	Euploid	Euploid
6	dup(12)(q13.11q13.3)(∼11Mb,∼30%)	Balanced	NA	Euploid	Euploid	Euploid
7	del(7)(p14.3p12.3)(∼17Mb,∼31%)	Balanced	NA	Euploid	Euploid	Euploid
8	del(8)(q24.22q24.3)(∼13Mb,∼31%)	Balanced	NA	Euploid	del(16)(p13.3)(∼4.80 Mb)	Euploid
9	dup(3)(p26.3p24.2)(∼25Mb,∼31%)	Balanced	NA	dup(3)(p26.3p21.1)(∼54.00 Mb,∼36%),+5(∼37%)	−Y(∼33%)	Euploid
10	dup(11)(p15.1p11.2)(∼27.80 Mb,∼31%)	Balanced	NA	Euploid	Euploid	Euploid
11	del(13)(q32.1q34)(∼19.77 Mb,∼34%)	Paternal	Mitosis	del(13)(q31.3q34)(∼20.97 Mb,∼50%)	del(13)(q31.3q34)(∼20.57 Mb,∼39%)	Euploid
12	−5(∼34%)	Paternal	Mitosis	Euploid	Euploid	Euploid
13	del(22)(q11.1q12.3)(∼21Mb,∼45%)	Balanced	NA	Euploid	−22(∼47%)	Euploid
	dup(22)(q13.1q13.32)(∼12Mb,∼45%)	Balanced	NA			
14	del(15)(q11.2q13.3)(∼10Mb,∼30%)	Balanced	NA	del(2)(q32.3q37.3)(∼47.60 Mb)	del(2)(q32.3q37.3)(∼47.60 Mb,∼47%),del(4)(p16.3p15.32)(∼16.00 Mb,∼40%)	del(2)(q32.3q37.3)(∼47.60 Mb,∼38%)
	del(2)(q32.3q37.3)(∼47.6 Mb,∼55%)	Paternal	Mitosis			
15	dup(20)(p12.2p11.21)(∼15Mb,∼35%)	Balanced	NA	del(20)(p13p11.1)(∼26.00 Mb,∼30%)	Euploid	del(20)(p13p11.1)(∼26.00 Mb,∼30%)
	del(8)(pterp12)(∼36Mb,∼48%)	Paternal	Mitosis			
16	del(7)(q11.22q36.3)(∼90Mb,∼51%)	Paternal	Mitosis	Euploid	dup(7)(q11.22q36.3)(∼89.94 Mb,∼46%)	Euploid
17	dup(21)(q11.2q22.3)(∼30.60 Mb,∼32%)	Balanced	NA	Euploid	Euploid	Euploid
	+10(∼41%)	Balanced	NA			
	dup(3)(p26.3q21.2)(∼124.80 Mb,∼51%)	Balanced	NA			
	del(1)(p36.32p21.3)(∼93.20 Mb,∼61%)	Paternal	Mitosis			
18	del(4)(q32.1q35.2)(∼34.00 Mb,∼61%)	Paternal	Mitosis	Euploid	Euploid	Euploid
19	−18(∼61%)	Maternal	Mitosis	Euploid	−18(∼30%)	Euploid
20	−3(∼64%)	Paternal	Mitosis	dup(3)(p26.3q26.31)(∼173.80 Mb),del(3)(q26.31q29)(∼24.40 Mb)	−3	del(3)(p26.3q26.31)(∼173.60 Mb,∼33%),del(3)(q26.31q29)(∼24.60 Mb)
21	del(13)(q21.1q34)(∼59.57 Mb,∼30%)	Balanced	NA	+16	+16	+16
	dup(12)(p13.33p11.1)(∼34.40 Mb,∼31%)	Balanced	NA			
	+16(∼64%)	Maternal	Meiosis			
22	del(16)(p13.2p12.3)(∼12Mb,∼30%)	Balanced	NA	+13, +9(∼30%)	+13	+13
	+13(∼67%)	Maternal	Meiosis			
23	+19(∼60%)	Maternal	Meiosis	+19, del(15)(q25.3q26.3)(∼14.53 Mb,∼39%)	+19(∼66%)	+19
24	+19p(∼54%)	Maternal	Meiosis	dup(19)(p13.3p12)(∼18.00 Mb,∼56%),dup(19)(p12p11)(∼4.20 Mb),dup(19)(q11q13.43)(∼31.13 Mb,∼60%)	dup(19)(p13.3p11)(∼23.00 Mb,∼61%),dup(19)(q11q13.11)(∼5.40 Mb),dup(19)(q13.11q13.43)(∼25.93 Mb,∼61%)	+19
	+19q(∼62%)	Maternal	Meiosis			

Note: TE, trophectoderm; NA, not applicable.

### Maternal meiotic errors dominate in aneuploid embryos

The 187 aneuploid embryos carried a total of 262 chromosomal aberrations, including 84 monosomies, 83 trisomies, 55 segmental deletions, and 40 segmental duplications (Fig. 3e and Supplementary Fig. S7a). Among them, 23 segmental deletions or duplications were excluded due to insufficient SNP information. Analysis of 167 monosomies and trisomies showed a striking predominance of maternal origin, with 90.4% (151/167) derived from maternal errors, 9.0% (15/167) from paternal errors, and 0.6% (1/167) showing balanced inheritance (Fig. 3e and Supplementary Fig. S7b). For the 72 analyzable segmental aberrations, 18.1% (13/72) showed balanced inheritance, 48.6% (35/72) were of maternal origin, and 33.3% (24/72) were of paternal origin. The 14 balanced CNVs identified in our study were predominantly associated with data quality warnings during the amplification and detection processes. Further analysis of cell-division errors demonstrated that 96.4% (160/166) of monosomies and trisomies with parental bias originated from meiotic errors, whereas only 69.5% (41/59) of segmental deletions and duplications were of meiotic origin (Fig. 3e and Supplementary Fig. S7c). A particularly notable finding was that 88.6% (147/166) of monosomies and trisomies resulted from maternal meiotic errors, which aligns with previous reports (Supplementary Fig. S7d) (Rana *et al.*, 2023).

Re-biopsies of 14 donated aneuploid embryos validated the analysis results of PH-trace. As shown in Table 1 and Supplementary Table S5, the 14 aneuploid embryos carried 21 CNVs, including 13 meiosis-derived, 5 mitosis-derived, and 3 false-positives. The results of reexamination showed that meiosis-derived chromosomal aberrations were evenly distributed in TE and ICM, while mitosis-derived aberrations exhibited a patchy distribution pattern (Supplementary Fig. S3 and Supplementary Table S5). The two aneuploid embryos carrying three false-positive CNVs were re-identified as euploid (Table 1 and Supplementary Fig. S6b). These results further confirmed the significance and reliability of PH-trace application in PGT-A.

### Distinct origins and characteristics of CNVs in aneuploid-mosaic embryos

**Figure 4.**
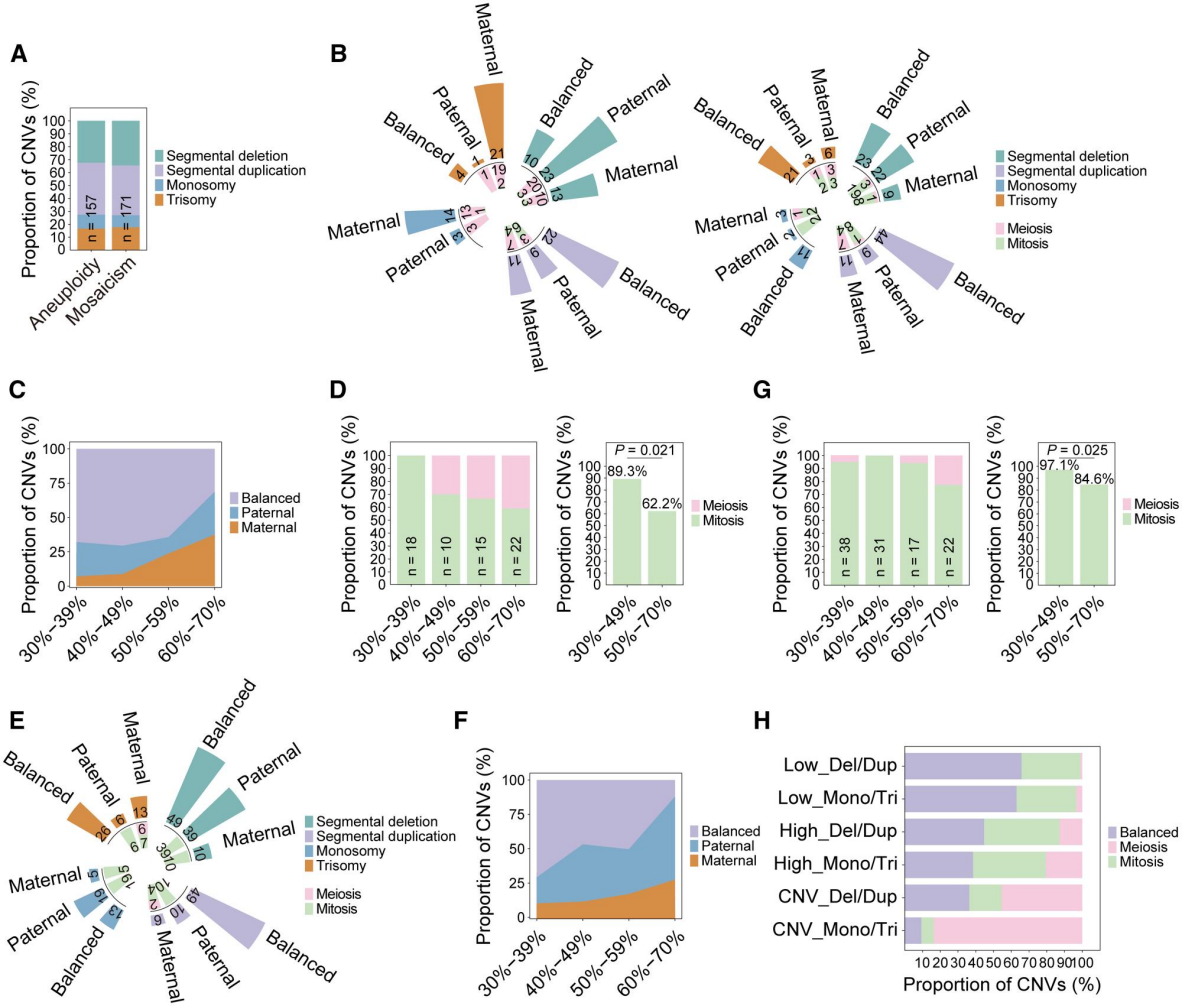
**Results of parental and cell-division origin analyses of aneuploid-mosaic and mosaic embryos.** (**A**) Overview of aneuploid and mosaic CNVs identified in aneuploid-mosaic embryos. (**B**) Results of parental and cell-division origin analysis of CNVs in aneuploid-mosaic embryos. Left: results of aneuploid CNVs. Right: results of mosaic CNVs. (**C**) Distribution of parental origin in CNVs with different mosaicism ratios from aneuploid-mosaic embryos. (**D**) Distribution of cell-division origin in CNVs with different mosaicism ratios from aneuploid-mosaic embryos. Low-ratio mosaicism is more likely to originate from mitotic errors than the high-ratio mosaicism (89.3% vs 62.2%, *P *= 0.021). (**E**) Results of parental and cell-division origin analysis of CNVs in mosaic embryos. (**F**) Distribution of parental origin in CNVs with different mosaicism ratios from mosaic embryos. (**G**) Distribution of cell-division origin in CNVs with different mosaicism ratios from mosaic embryos. Both low- and high-ratio mosaic CNVs are more likely to come from mitotic errors (97.1% vs 84.6%, *P *= 0.025). (**H**) The proportions of CNVs with balanced parental and mitotic/meiotic origin in aneuploid-mosaic and mosaic embryos. A range of 30–49% is defined as low-level mosaicism, while a range of 50–70% is defined as high-level mosaicism. Low_Del/Dup, low-level mosaic deletion and duplication; Low_Mono/Tri, low-level mosaic monosomy and trisomy; High_Del/Dup, high-level mosaic deletion and duplication; High_Mono/Tri, high-level mosaic monosomy and trisomy; CNV_Del/Dup, aneuploid deletion and duplication; CNV_Mono/Tri, aneuploid monosomy and trisomy; CNVs, copy-number variations.

Next, we applied the PH-trace strategy to reanalyze the aneuploid-mosaic embryos (Fig. 1). The 94 aneuploid-mosaic embryos carried 157 aneuploid CNVs (43 whole-chromosome aberrations and 114 segmental aberrations) and 171 mosaic CNVs (46 whole-chromosome aberrations and 125 segmental aberrations) (Fig. 4a and Supplementary Fig. S8a). For aneuploid CNVs, the majority of monosomies and trisomies (81.4%, 35/43) originated from maternal errors, consistent with the findings in aneuploid embryos (Fig. 4b, left panel and Supplementary Fig. S8b). Notably, meiotic errors, especially maternal meiotic errors, accounted for the main proportion (Supplementary Fig. S8c and d). After excluding 26 aneuploid deletions or duplications with insufficient SNP information, 36.4% (32/88) of deletions and duplications exhibited a balanced pattern (Fig. 4b, left panel and Supplementary Fig. S8b). Meiotic errors accounted for the majority of deletions and duplications and exhibited no preference for parental sources (Supplementary Fig. S8c and d). These findings reveal a significantly higher maternal meiotic contribution to monosomies and trisomies than to deletions and duplications (82.1% vs 30.4%, *P *< 0.001) (Fig. 4b, left panel and Supplementary Fig. S8d).

The mosaic CNVs exhibited even more pronounced differences. Both whole-chromosome and segmental mosaic CNVs showed a remarkable balanced pattern, accounting for 69.5% (32/46) and 56.8% (67/118) (Fig. 4b, right panel and Supplementary Fig. S8e). Strikingly, 76.5% (39/51) of segmental mosaic CNVs originated from mitotic errors, compared to 64.3% (9/14) of whole-chromosome mosaic CNVs (Fig. 4b, right panel and Supplementary Fig. S8f). The mosaic ratio was inversely related to the proportion of false-positive CNVs (*P *= 0.003; Fig. 4c), and mitotic origin was predominantly observed in low-ratio mosaics (Fig. 4d). Mosaic monosomies and trisomies did not exhibit the pronounced maternal meiotic contribution that was observed in aneuploid CNVs (Supplementary Fig. S8g). One aneuploid-mosaic embryo carrying two mitosis-derived CNVs and one balanced CNV was re-biopsied and subsequently reclassified as euploid (Table 1). These findings showed that the mosaic events exhibited higher rates of false-positives and mitotic origin compared with aneuploid events, suggesting fundamentally different formation mechanisms between these abnormality classes.

### Distinct features of segmental versus whole-chromosome CNVs in mosaic embryos

Among the 173 mosaic embryos carrying 247 mosaic CNVs, segmental aberrations predominated (66.8%, 165/247), and two CNVs were excluded due to insufficient SNP information (Supplementary Fig. S9a and b). Segmental aberrations presented a higher rate of false-positive (60.1% vs 47.6%, *P *= 0.076), even without statistical significance, and a lower maternal origin bias (9.8% vs 21.9%, *P *= 0.012) compared with whole-chromosome aberrations (Fig. 4e and Supplementary Fig. S9c). Mitotic errors overwhelmingly accounted for mosaic CNVs and were particularly prevalent in segmental aberrations compared to whole-chromosome aberrations (96.9% vs 86.0%, *P = *0.057) (Fig. 4e and Supplementary Fig. S9d). Paternal mitotic errors were the predominant source of both segmental and whole-chromosome aberrations, accounting for 75.4% and 58.1%, respectively (Supplementary Fig. S9e). In addition, the mosaic ratio significantly influenced CNV characteristics. The proportion of false-positive CNVs gradually decreased with increasing levels of mosaicism (*P *< 0.001) (Fig. 4f and Supplementary Table S6). Notably, low-ratio mosaics showed a significantly higher mitotic origin than high-ratio mosaics (97.1% vs 84.6%, *P = *0.025) (Fig. 4g and Supplementary Table S6).

Validation studies using 21 mosaic embryos further confirmed the reliability of the method (Table 1). 94.1% (16/17) of the CNVs predicted to be false-positive were not detected upon re-biopsy (Table 1), supporting the technical artifacts. Of the 14 CNVs predicted to be true, 71.4% (10/14) were re-detected. The remaining CNVs, which were not detected upon re-biopsy, were all of mitotic origin and may have been affected by sampling location. In contrast, all five CNVs of meiotic origin were successfully confirmed.

In summary, the comprehensive analysis of 157 aneuploid CNVs and 418 mosaic CNVs showed that false-positives and mitotic errors were more prevalent in low-ratio chromosomal aberrations, whereas meiotic errors were more frequent in high-ratio aberrations, particularly in monosomies and trisomies (Fig. 4h). For mosaic embryo transfer, we recommend prioritizing embryos with lower mosaic ratios (<50%) and segmental rather than whole-chromosome aberrations, as these characteristics correlate with higher rates of false-positives and mitotic errors.

### Clinical outcomes of mosaic transfers

**Figure 5.**
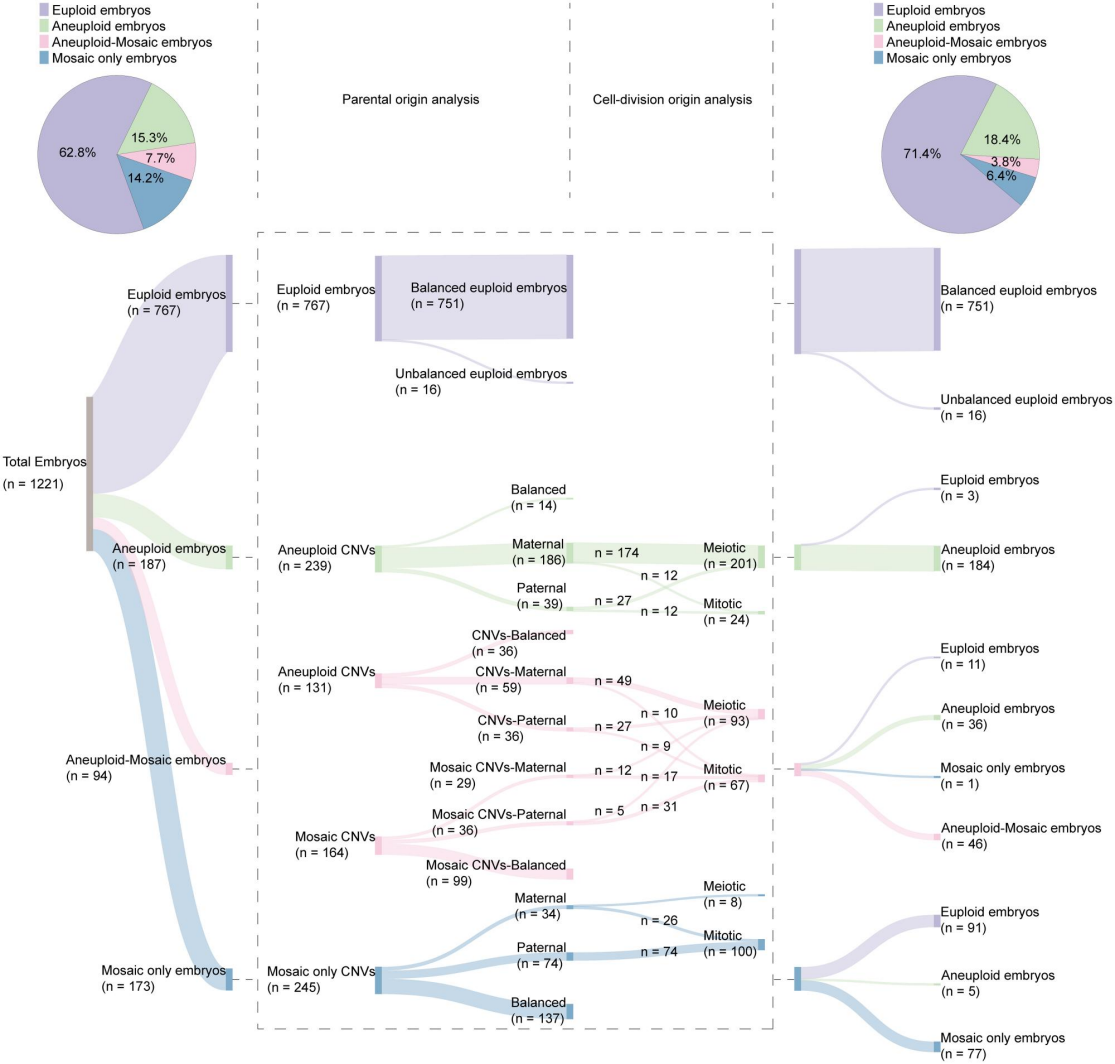
**Results of the analysis of 1221 embryos.** The parental and cell-division origin analysis identified 16 euploid embryos with uniparental disomy or heteroploidy and 3 euploid embryos with data quality warnings during amplification and detection from 187 aneuploid embryos. Among 94 aneuploid-mosaic embryos, 11 were identified as euploid embryos, 36 were identified as aneuploid embryos, and one was identified as a mosaic embryo only. Among the 173 mosaic embryos, 91 euploid embryos and 5 aneuploid embryos were identified, respectively. After data analysis, the proportion of mosaic embryos decreased from 14.2% to 6.4% and the proportion of euploid embryos increased from 62.8% to 71.4%. A total of 58 CNVs were excluded due to a limited number of single-nucleotide polymorphisms (SNPs), including 23 from aneuploid embryos, 33 from aneuploid-mosaic embryos, and 2 from mosaic embryos. CNVs, copy-number variations.

As shown in Fig. 5, reanalysis of 1221 embryos using the PH-trace strategy detected heteroploidy among euploid embryos and rectified prior misclassifications of aneuploid, aneuploid-mosaic, and mosaic embryos. Specifically, 52.6% (91/173) of embryos initially classified as mosaic were reclassified as euploid, which reduced the mosaicism detection rate from 14.2% to 6.4%. Additionally, the reidentification of 105 euploid embryos from the aneuploid, aneuploid-mosaic, and mosaic categories increased the usable embryo rate from 62.8% to 71.4%, representing an 8.6% increase. These results demonstrate that the refined approach significantly reduces false-positives and enhances the diagnostic accuracy of PGT-A.

Clinical outcomes from 19 transferred mosaic embryos carrying 25 CNVs were reassuring, with a clinical pregnancy of 52.6% and a live birth rate of 47.4% (Table 2). Among the 9 mosaic embryos that resulted in live births, 66.7% (6/9) were false-positives, 22.2% (2/9) originated mitotically, and 11.1% (1/9) were of uncertain origin. Crucially, the 25 mosaic CNVs, including 17 false-positive, 7 mitosis-derived and one of uncertain origin, were not detected again in either amniotic fluid or miscarriage tissue samples (Table 2 and Supplementary Fig. S10). These findings demonstrate that low-ratio segmental mosaics have a high likelihood of being false-positives, and mitotic errors may undergo embryonic self-correction. Our reanalysis strategy effectively reduces the unnecessary discard of embryos.

**Table 2.** Pregnancy outcomes of mosaic embryos.

ID	PGT-A	Parental origin	Cell-division origin	Prenatal diagnosis	Reproductive outcome
1	del(16)(p13.3p12.3) (∼13Mb, ∼30%)	Balanced	NA	Euploid	Live birth
2	del(8)(q21.3q24.3)(∼58Mb, ∼32%)	Balanced	NA	Euploid	Live birth
3	dup(15)(q22.31q25.1)(∼13Mb, ∼32%)	Balanced	NA	Euploid	Live birth
4	−4p(50%)	Balanced	NA	Euploid	Live birth
	−4q(∼50%)	Balanced	NA		
5	dup(22)(q11.1q13.1)(∼21.00 Mb,∼38%)	Balanced	NA	Euploid	Live birth
	+8(∼54%)	Balanced	NA		
6	del(6)(q22.1q25.3)(∼41Mb, ∼45%)	Balanced	NA	Euploid	Live birth
7	del(16)(p13.2p12.1)(∼18Mb, ∼44%)	Maternal	Mitosis	Euploid	Live birth
8	del(8)(q11.1q12.1)(∼12.20 Mb,∼33%)	Uncertain	NA	Euploid	Live birth
9	del(8)(q24.22q24.3)(∼13Mb,∼31%)	Balanced	NA	NA	Failed
10	dup(17)(q12q21.32)(∼13Mb, ∼31%)	Balanced	NA	NA	Failed
11	+20(∼34%)	Balanced	NA	NA	Failed
12	del(2)(q31.3q33.1)(∼18Mb ,∼33%)	Balanced	NA	NA	Failed
	del(2)(q22.1q23.2)(∼14Mb, ∼35%)	Balanced	NA		
13	−8p(∼40%)	Balanced	NA	NA	Failed
14	−3p(∼32%)	Balanced	NA	NA	Failed
15	del(3)(p26.3p14.1)(∼65Mb, ∼34%)	Paternal	Mitosis	Euploid	Early miscarriage
16	del(18)(q12.1q23)(∼49Mb, ∼37%)	Paternal	Mitosis	NA	Failed
17	+18p(∼30%)	Balanced	NA	NA	Failed Failed
	−7p(∼34%)	Balanced	NA		
	−7q(∼38%)	Maternal	Mitosis		
18	del(15)(q11.2q22.2)(∼38Mb, ∼35%)	Maternal	Mitosis	NA	Failed
	del(15)(q24.3q26.3)(∼24Mb, ∼38%)	Maternal	Mitosis		
19	−8(∼50%)	Paternal	Mitosis	Euploid	Live birth

Note: PGT, preimplantation genetic testing; NA, not applicable.

## Discussion

Our study establishes PH-trace analysis as a robust method to resolve diagnostic uncertainty in PGT-A. By quantifying allelic imbalance through the novel ‘uneven score’ metric, we reclassified 52.6% of mosaic embryos as euploid, reduced the mosaicism prevalence from 14.2% to 6.4%, and increased the proportion of usable embryos by 8.6%. This recalibration resolves the marked discrepancy in mosaicism rates between PGT-A and prenatal diagnosis, highlighting the attribution of technical artifacts rather than true biological incidence. Crucially, PH-trace overcomes the limitations of conventional intermediate copy-number thresholds, which yield highly variable mosaicism detection rates across laboratories.

The cell-division origin analysis furtherly revealed fundamental biological differences between aneuploid CNVs and mosaic CNVs. For the aneuploid CNVs, they predominantly originated from maternal meiotic errors, particularly in cases involving whole-chromosome aberrations. These findings are consistent with previous reports, indicating that aneuploidy caused by meiosis errors is more common in human oocytes than in sperm, and its incidence increases with advanced maternal age (Gruhn *et al.*, 2019; Charalambous *et al.*, 2023; Rana *et al.*, 2023). Mosaic CNVs predominantly arise from mitotic errors, exhibit frequent false-positives, and show no association with advanced maternal age (Taylor *et al.*, 2014; Marin *et al.*, 2021; Ren *et al.*, 2022; Cascales *et al.*, 2023). Moreover, mitotic errors occurring at very early developmental stages or the mitotic rescue of a meiotic error often result in generalized mosaicism, while mitotic errors arising in specific cell lineages typically lead to confined mosaicism (Taylor *et al.*, 2014). This mechanistic divergence explains their contrasting clinical outcomes, as aneuploid embryos consistently develop into affected fetuses, while transferred mosaic embryos often lack the originally detected aberrations. Moreover, advanced single-cell karyotyping revealed that up to 80–100% of blastocysts contain minor (<20%) aneuploid cell lines, a finding obscured by the 20–30% detection threshold of conventional bulk multicellular TE sequencing (Chavli *et al.*, 2024; Robertson and Richards, 2024; Zhai *et al.*, 2024). Mosaicism may be a common phenomenon in human early embryos, and increasing evidence also indicates human fetuses and even adults exhibit a degree of tolerance to aneuploidy (McConnell *et al.*, 2013; Knouse *et al.*, 2014; Zamani Esteki *et al.*, 2019; Yang *et al.*, 2021). These findings support the transfer of certain mosaic embryos when no euploid embryos are available, although rigorous counseling on residual risks remains essential.

Professional societies such as the American Society for Reproductive Medicine (ASRM), ESHRE, and the Preimplantation Genetic Diagnosis International Society (PGDIS) recommend that selection of mosaic embryos for transfer should be based on a comprehensive assessment of embryo morphology, the level and type of mosaicism, and the number of specific chromosomes involved (De Rycke *et al.*, 2022; Leigh *et al.*, 2022; Practice Committees of the American Society for Reproductive Medicine and the Genetic Counseling Professional Group, 2023). Embryos with low-level or segmental mosaicism are often regarded as preferable candidates for transfer (Leigh *et al.*, 2022). In this study, we further exposed critical differences between whole-chromosome and segmental aberrations. Unlike whole-chromosome aberrations, which predominantly originate from maternal meiotic errors, segmental aberrations exhibit a higher false-positive rate and are more frequently of mitotic origin. Significant differences were also observed between low-ratio and high-ratio mosaicism, with low-ratio mosaicism more frequently exhibiting false-positives and being of mitotic origin. These findings further emphasize the transfer prioritization of low-ratio segmental mosaic embryos in clinical practice. Thus, the PH-trace strategy directly addresses three major challenges faced by clinicians in the management of mosaic embryos: (1) the inability to distinguish technical artifacts from true biological signals, (2) uncertainty regarding the origin of cell-division errors, and (3) conservative transfer practices based on unvalidated risks.

Our findings suggest that mosaic CNVs could undergo self-correction during embryonic development. Supporting this, mosaic embryos cultured *in vitro* until Day 12 were found to convert to euploid status, and the proportion of mosaicism in human embryonic stem cells derived from the ICM was observed to gradually decline over time (Popovic *et al.*, 2019; Ren *et al.*, 2022). Currently, several major models have been proposed to explain the self-correction of mosaic embryos, including the clonal clearance model, exclusion model, and selective distribution model (Coticchio *et al.*, 2021; Zhang and Zheng, 2024). For the clonal clearance model, embryos eliminate aneuploid cells via apoptosis or elevated autophagy levels during the post-implantation stage, while euploid cells compensate for cell loss through increased proliferation (Bolton *et al.*, 2016; Singla *et al.*, 2020). For the exclusion model, abnormal chromosomes can be expelled during embryonic development through the extrusion of cell fragments into the perivitelline space or unequal cleavage (Zhan *et al.*, 2016; Daughtry *et al.*, 2019; Orvieto *et al.*, 2020). For the selective distribution model, studies have shown that aneuploid cells gradually accumulate in the TE after implantation, indicating a dynamic and asymmetrical distribution of aneuploid cells between the TE and ICM during embryonic development (Zhou *et al.*, 2019; Starostik *et al.*, 2020). This phenomenon may be explained by the preferential elimination of abnormal cells in the ICM, along with the greater tolerance of TE cells to aneuploidy (Bolton *et al.*, 2016; Yang *et al.*, 2021).

The limitations of this study include the dependency of PH-trace on sufficiently informative SNPs (>30 pre-aberration), which may exclude smaller CNVs, and the lack of long-term neonatal follow-up data. Nevertheless, our validation confirmed that 94.1% of predicted false-positives were not detectable upon re-biopsy, while 71.4% of true mosaic cases persisted. Secondly, although PGT-M + A was performed by the same testing institution, this retrospective study may be subject to unmeasured confounding, potentially influencing the detection rate of false-positive mosaicism. Moreover, this study focused exclusively on PGT-A + M cycles; however, our data demonstrated comparable mosaic embryo detection rates between PGT-A + M and PGT-A-only cycles. Future studies should aim to establish standardized SNP density requirements across different types of chromosomal aberrations. In addition, large-scale prospective studies are warranted to validate the utility of this method in improving the clinical application of PGT-A and to monitor the long-term development outcomes of offspring derived from the transfer of embryos with mitotic errors.

In conclusion, our study suggests that the implementation of PH-trace could improve the clinical application of PGT-A by: (1) reducing the incidence of false mosaic embryos, improving the accuracy of predicting mosaicism, and increasing the rate of euploid embryos; and (2) clarifying the cell-division origin (meiotic/mitotic) to support evidence-based embryo selection and ultimately improve reproductive outcomes. Future large-scale prospective studies are planned to validate the utility of this method in the clinical application of PGT-A.

## Supplementary Material

hoaf075_Supplementary_Data

## Data Availability

The data in this article will be shared upon reasonable request to the corresponding author.
