# Supplementary material for: Parental and cell-division origin analysis to reduce false-positives in mosaic embryos for preimplantation genetic testing
Source: Hum Reprod Open. 2025 Dec 1;2025(4):hoaf075. doi: 10.1093/hropen/hoaf075 (PMC12714397; doi:10.1093/hropen/hoaf075)
Supplement: hoaf075_Supplementary_Data [file hoaf075_supplementary_data.docx]

**SUPPLEMENTARY TABLES AND FIGURES**

Parental and Cell-Division Origin Analysis to Reduce False Positives in Mosaic Embryos for Preimplantation Genetic Testing

Supplementary Table S1 The proportion of mosaic embryo cycles in different PGT cycles.

Supplementary Table S2 Distribution of PGT and prenatal diagnosis results.

Supplementary Table S3. Information on euploid transfer with subsequent prenatal diagnosis of aneuploidy

Supplementary Table S4 Distribution of mosaic ratio and variation types in mosaic CNVs.

Supplementary Table S5 The results of trophectoderm and ICM cell biopsies from aneuploid embryos

Supplementary Table S6 Analysis of different mosaicism ratios from mosaic embryos.

Supplementary Figure S1. Parental origin analysis of chromosomal aberrations.

Supplementary Figure S2. Cell-division origin analysis of chromosomal aberrations.

Supplementary Figure S3. Re-biopsies for aneuploid embryos positive controls.

Supplementary Figure S4. Prenatal diagnosis results of mosaic and aneuploid embryo transfers after PGT treatment.

Supplementary Figure S5. Chromosome ideograms of mosaic CNVs identified in PGT treatment and prenatal diagnosis of natural pregnancies.

Supplementary Figure S6. The analysis results of euploid and aneuploid embryos.

Supplementary Figure S7. Analysis of parental and cell-division origins in aneuploid embryos.

Supplementary Figure S8. Analysis of parental and cell-division origins in aneuploid-mosaic embryos.

Supplementary Figure S9. Analysis of parental and cell-division origins in mosaic embryos.

Supplementary Figure S10. Prenatal diagnosis results of mosaic embryo transfers in PGT-A+M treatment cycles.

**Supplementary Table S1. The proportion of mosaic embryo cycles in different PGT cycles**

| **PGT Type** | **Cycle Type** | **Num of cycles** | **Proportion** |
| --- | --- | --- | --- |
| PGT-A only | Euploid cycle | 923 | 75.6% |
|  | Mosaic cycle | 78 | 6.4% |
|  | Aneuploid cycle | 219 | 18.0% |
|  | Total | 1220 | 100.0% |
| PGT-A +SR | Euploid cycle | 523 | 67.7% |
|  | Mosaic cycle | 69 | 8.9% |
|  | Aneuploid cycle | 181 | 23.4% |
|  | Total | 773 | 100.0% |
| PGT-A +M | Euploid cycle | 433 | 79.6% |
|  | Mosaic cycle | 55 | 10.1% |
|  | Aneuploid cycle | 56 | 10.3% |
|  | Total | 544 | 100.0% |
| Total PGT-A | Euploid cycle | 1879 | 74.0% |
|  | Mosaic cycle | 202 | 8.0% |
|  | Aneuploid cycle | 456 | 18.0% |
|  | Total | 2537 | 100.0% |

Note: Euploid cycles were defined as those containing euploid embryos; mosaic cycles as those without available euploid embryos but containing mosaic embryos; and aneuploid cycles as those containing only aneuploid embryos. PGT-A, preimplantation genetic testing for aneuploidy; PGT-M, preimplantation genetic testing for monogenic disorders; PGT-SR, preimplantation genetic testing for chromosomal structural rearrangements.

**Supplementary Table S2. Distribution of PGT and prenatal diagnosis results**

| **Item** | **Characteristics** | **Euploid (%)** | **Mosaic (%)** | **Aneuploid-Mosaic (%)** | **Aneuploid (%)** | **Total (%)** |
| --- | --- | --- | --- | --- | --- | --- |
| PGT diagnosis | PGT-A only | 2324 (54.9) | 562 (13.3) | 145 (3.4) | 1204 (28.4) | 4235 (100) |
|  | PGT-A+SR | 1064 (38.3) | 261 (9.4) | 174 (6.3) | 1278 (46.0) | 2777 (100) |
|  | PGT-A+M | 1231 (60.0) | 285 (14.0) | 122 (5.9) | 412 (20.1) | 2050 (100) |
|  | Total PGT-A | 4619 (51.0) | 1108 (12.2) | 441 (4.9) | 2894 (31.9) | 9062 (100) |
| Prenatal diagnosis | Natural pregnancy | 6290 (80.1) | 74 (0.9) | 29 (0.4) | 1463(18.6) | 7856 (100) |
|  | Traditional IVF treatment | 482 (88.1) | 1 (0.2) | 1 (0.2) | 63 (11.5) | 547 (100) |
|  | Euploid embryo transfer | 204 (92.3) | 2 (0.9) | 1 (0.5) | 14 (6.3) | 221 (100) |
|  | Mosaic embryo transfer | 19 (100) | 0 (0) | 0 (0) | 0 (0) | 19 (100) |
|  | Aneuploid embryo transfer | 0 (0) | 0 (0) | 0 (0) | 2 (100) | 2(100) |

Note: PGT-A, preimplantation genetic testing for aneuploidy; PGT-M, preimplantation genetic testing for monogenic disorders; PGT-SR, preimplantation genetic testing for chromosomal structural rearrangements.

**Supplementary Table S3. Information on euploid transfer with subsequent prenatal diagnosis of aneuploidy**

| **ID** | **PGT diagnosis** | **Prenatal karyotype** | **Prenatal CNV sequence** | | | | | **Other information** |
| --- | --- | --- | --- | --- | --- | --- | --- | --- |
|  |  |  | **Chromosome position*** | **CNV size** | **Deletion/Duplication** | **Clinical significance** | **Origin** |  |
| 1 | Euploid | 46,XN | chr5:g.36670001-45900000 | 9.23 Mb | 47% mosaic duplication | Uncertain | *De novo* | Unnoted in PGT |
|  |  |  | chr5:g.45900001-46300000 | 400 kb | Duplication | Uncertain | *De novo* | Unnoted in PGT |
| 2 | Euploid | 46,XN,?ins(4)(q21.1p15.3p15.2) | chr4:g.13460001_14270000 | 810 kb | Deletion | Uncertain | Mother | Noted in PGT |
|  |  |  | chr4:g.21450001_22230000 | 780 kb | Deletion | Uncertain | Mother | Noted in PGT |
| 3 | Euploid | 46,XN | chr5:g.163440001_164880000 | 1.44 Mb | Duplication | Uncertain | NA | Noted in PGT |
| 4 | Euploid | 46,XN | chr4:g.188110001_189500000 | 1.39 Mb | Duplication | Uncertain | Father | Noted in PGT |
| 5 | Euploid | 46,XN | chr16:g.78370001_81720000 | 3.35 Mb | Duplication | Uncertain | NA | Noted in PGT |
| 6 | Euploid | 46,XN,t(12;13)(q24.3;q12) | chr13:g.22610001_23240000 | 630 kb | Deletion | Uncertain | NA | Unnoted in PGT |
| 7 | Euploid | 46,XN | chrX:g.394824_744821 | 350 kb | Deletion | Pathogenic | Father | Noted in PGT |
| 8 | Euploid | 46,XN,inv(9)(p12q13) | chr4:g.70001_220000 | 150 kb | Deletion | Uncertain | Mother | Unnoted in PGT |
| 9 | Euploid | 45,XN,der(14;21)(q10;q10) | chr17:g.17530001_17640000 | 110 kb | Duplication | Uncertain | *De novo* | Unnoted in PGT |
| 10 | Euploid | 46,XN | chr2:g.185860001_186360000 | 500 kb | Deletion | Uncertain | Father | Unnoted in PGT |
| 11 | Euploid | 46,XN | chr8:g.16070001_17210000 | 1.14 Mb | Duplication | Uncertain | Father | Noted in PGT |
| 12 | Euploid | 46,XN | chr15:g.22750001_23090000 | 340 kb | Deletion | Pathogenic | Father | Unnoted in PGT. The estimated penetrance is about 10.4%. |
| 13 | Euploid | 46,XN | chr10:g.57870001_59270000 | 1.4 Mb | Deletion | Uncertain | NA | Noted in PGT |
| 14 | Euploid | 46,XN | chr8:g.2350001_5780000 | 3.43 Mb | Duplication | Uncertain | NA | Noted in PGT |
| 15 | Euploid | 46,XN | chr2:g.59900001_61710000 | 1.81 Mb | Duplication | Uncertain | *De novo* | Noted in PGT |
| *: The reference genome was hg19. NA: not available. Traditional PGT-A detection only report chromosomal abnormalities ≥ 4 MB, while prenatal diagnosis report chromosomal abnormalities ≥ 100 kb. | | | | | | | | |

**Supplementary Table S4. Distribution of mosaic ratio and variation types in mosaic CNVs**

| **Item** | **Groups** | **PGT-A (%)** | **Prenatal testing from natural pregnancy (%)** |
| --- | --- | --- | --- |
| Mosaic ratio | 10%-29% | - | 49 (41.5) |
|  | 30%-39% | 735 (46.5) | 15 (12.7) |
|  | 40%-49% | 435 (27.5) | 15 (12.7) |
|  | 50%-59% | 218 (13.8) | 10 (8.5) |
|  | 60%-70% | 192 (12.2) | 15 (12.7) |
|  | 71%-90% | - | 14 (11.9) |
|  | Total | 1580 (100) | 118 (100) |
| CNV type | Segmental deletion | 573 (36.3) | 13 (11.0) |
|  | Segmental duplication | 345 (21.8) | 23 (19.5) |
|  | Monosomy | 308 (19.5) | 42 (35.6) |
|  | Trisomy | 354 (22.4) | 40 (33.9) |
|  | Total | 1580 (100) | 118 (100) |

Note: The reported range of mosaicism is 30%-70% in PGT-A, whereas it is 10%-90% in prenatal diagnosis. PGT-A, preimplantation genetic testing for aneuploidy; CNVs, copy-number variations; “-”, not applicable.

**Supplementary Table S5 The results of trophectoderm and ICM cell biopsies from aneuploid embryos**

| **ID** | **Clinical TE biopsy** | **Parental origins** | **Cell-division origins** | **Blinded analysis of re-biopsies** | | | | **Aneuploidy distribution** |
| --- | --- | --- | --- | --- | --- | --- | --- | --- |
|  |  |  |  | **TE_1** | **TE_2** | **TE_3** | **ICM** |  |
| 1 | +9 | Maternal | Meiosis | +9 | +9 | +9 | +9 | Uniform |
|  | +18 | Maternal | Meiosis | +18 | +18 | +18 | +18 | Uniform |
|  | +21 | Maternal | Meiosis | +21 | +21 | +21 | +21 | Uniform |
| 2 | +2 | Maternal | Meiosis | +2 | +2 | +2 | +2 | Uniform |
| 3 | -13 | Maternal | Meiosis | -13 | -13 | -13 | -13 | Uniform |
|  | -19 | Maternal | Meiosis | -19 | -19 | -19 | -19 | Uniform |
| 4 | -22 | Maternal | Meiosis | -22 | -22 | -22 | -22 | Uniform |
| 5 | +11 | Maternal | Meiosis | +11 | +11 | +11 | +11 | Uniform |
|  | +21 | Maternal | Meiosis | +21 | +21 | +21 | +21 | Uniform |
| 6 | del(8)(p23.3p11.22)(~38.80Mb) | Paternal | Meiosis | del(8)(p23.3p11.1)(~43.60Mb) | del(8)(p23.3p11.22)(~39.00Mb) | del(8)(p23.3p11.1)(~43.60Mb) | del(8)(p23.3p11.22)(~38.80Mb) | Uniform |
| 7 | del(5)(q11.2q35.3)(~127.72Mb) | Maternal | Meiosis | -5 | del(5)(p15.33q11.2)(~53.00Mb,~30%), del(5)(q11.2q35.3)(~128.12Mb) | del(5)(q11.2q35.3)(~127.72Mb) | -5 | Uniform* |
| 8 | +9 | Maternal | Meiosis | +9 | +9 | +9 | +9 | Uniform |
|  | +15 | Maternal | Mitosis | - | - | - | - | Non-uniform |
| 9 | -16 | Maternal | Meiosis | -16 | -16 | -16 | -16 | Uniform |
|  | dup(4)(p16.3p11)(~49.20Mb) | Paternal | Mitosis | del(4)(p16.3p11)(~49.20Mb,~62%) | - | del(4)(p16.3p11)(~49.20Mb,~30%) | - | Non-uniform |
| 10 | del(13)(q31.1q34)(~29.97Mb) | Paternal | Mitosis | del(13)(q31.1q34)(~30.17Mb) | del(13)(q31.1q34)(~30.37Mb) | del(13)(q31.1q34)(~30.17Mb) | - | Non-uniform |
| 11 | dup(1)(q25.3q44)(~67.25Mb) | Paternal | Mitosis | - | - | - | - | Non-uniform |
| 12 | dup(11)(p15.5p12)(~36.20Mb) | Paternal | Mitosis | - | - | - | - | Non-uniform |

Note: TE, trophectoderm; ICM, inner cell mass; -, the corresponding chromosomal aberration was not detected in the respective re-biopsy; *, mitosis events may happen in chromosome 5.

**Supplementary Table S6 Analysis of different mosaicism ratios from mosaic embryos**

| **Parental and cell-division origins** | **Mosaicism ratios** | **Segmental aberrations** | **whole-chromosome abnormalities** | **Total** |
| --- | --- | --- | --- | --- |
| Parental origins | 30%-39% |  |  |  |
|  | Balanced (%) | 66 (76.7) | 24 (57.2) | 90 (70.3) |
|  | Maternal (%) | 6 (7.0) | 8 (19.0) | 14 (10.9) |
|  | Paternal (%) | 14 (16.3) | 10 (23.8) | 24 (18.8) |
|  | Total (%) | 86 (100) | 42 (100) | 128 (100) |
|  | 40%-49% |  |  |  |
|  | Balanced (%) | 15 (40.6) | 12 (57.1) | 27 (46.5) |
|  | Maternal (%) | 4 (10.8) | 3 (14.3) | 7 (12.1) |
|  | Paternal (%) | 18 (48.6) | 6 (28.6) | 24 (41.4) |
|  | Total (%) | 37 (100) | 21 (100) | 58 (100) |
|  | 50%-59% |  |  |  |
|  | Balanced (%) | 14 (56.0) | 3 (33.3) | 17 (50.0) |
|  | Maternal (%) | 4 (16.0) | 2 (22.2) | 6 (17.6) |
|  | Paternal (%) | 7 (28.0) | 4 (44.5) | 11 (32.4) |
|  | Total (%) | 25 (100) | 9 (100) | 34 (100) |
|  | 60%-70% |  |  |  |
|  | Balanced (%) | 3 (20.0) | 0 (0) | 3 (12.0) |
|  | Maternal (%) | 2 (13.3) | 5 (50.0) | 7 (28.0) |
|  | Paternal (%) | 10 (66.7) | 5 (50.0) | 15 (60.0) |
|  | Total (%) | 15 (100) | 10 (100) | 25 (100) |
|  | Total |  |  |  |
|  | Balanced (%) | 98 (60.1) | 39 (47.6) | 137 (55.9) |
|  | Maternal (%) | 16 (9.8) | 18 (21.9) | 34 (13.9) |
|  | Paternal (%) | 49 (30.1) | 25 (30.5) | 74 (30.2) |
|  | Total (%) | 163 (100) | 82 (100) | 245 (100) |
| Cell-division origins | 30%-39% |  |  |  |
|  | Mitosis (%) | 20 (100) | 16 (88.9) | 36 (94.7) |
|  | Meiosis (%) | 0 (0) | 2 (11.1) | 2 (5.3) |
|  | Total (%) | 20 (100) | 18 (100) | 38 (100) |
|  | 40%-49% |  |  |  |
|  | Mitosis (%) | 22 (100) | 9(100) | 31 (100) |
|  | Meiosis (%) | 0 (0) | 0 (0) | 0 (0) |
|  | Total (%) | 22 (100) | 9 (100) | 31 (100) |
|  | 50%-59% |  |  |  |
|  | Mitosis (%) | 10 (90.9) | 6 (100) | 16 (94.1) |
|  | Meiosis (%) | 1 (9.1) | 0 (0) | 1 (5.9) |
|  | Total (%) | 11 (100) | 6 (100) | 17 (100) |
|  | 60%-70% |  |  |  |
|  | Mitosis (%) | 11 (91.7) | 6 (60.0) | 17 (77.3) |
|  | Meiosis (%) | 1 (8.3) | 4 (40.0) | 5 (22.7) |
|  | Total (%) | 12 (100) | 10 (100) | 22 (100) |
|  | Total |  |  |  |
|  | Mitosis (%) | 63 (96.9) | 37 (86.0) | 100 (92.6%) |
|  | Meiosis (%) | 2 (3.1) | 6 (14.0) | 8 (7.4%) |
|  | Total (%) | 65 (100) | 43 (100) | 108 (100) |

**Supplementary Figure S1. Parental origin analysis of chromosomal aberrations.** (**a**) Homozygous SNPs with biallelic alleles in parental genomes, such as the reference allele AA and the alternative allele BB, were selected as informative SNPs for parental origin analysis. The possible inheritance patterns of monosomy and trisomy with different parental origins were shown. (**b**) The distribution of mBAF in five kinds of chromosomes, such as maternal monosomy/trisomy, paternal monosomy/trisomy, and balanced, were shown. mBAF was termed as the count of maternal allele as the numerator and the sum of maternal and paternal alleles as the denominator at the selected SNPs in (**a**). mBAF, maternal BAF; SNPs, single nucleotide polymorphisms.

**Supplementary Figure S2. Cell-division origin analysis of chromosomal aberrations.** (**a**) For maternal trisomy, SNPs with paternal homozygous and maternal heterozygous were selected to analyze the cell-division origin. As the synapsis and crossover of homologous chromosomes occurred in meiosis, the location of crossover and the presence or absence of heterozygosity in embryonic homologous chromosomes were referred to determining the period of abnormal occurrence, such as meiosis I, meiosis II, and mitosis. The F1 and F2 represent the two homologous chromosomes from the paternal origin, while M1 and M2 represent the two homologous chromosomes from the maternal origin. (**b**) Monosomy originating from meiotic errors displayed loss of heterozygosity, while monosomy originating from mitotic errors presented with heterozygosity, in the remaining chromosome. (**c**) The diagram of SNP distribution in trisomy originating from different cell-division phases. BPH: both parental homologs; SPH: single parental homolog; Cen: centromere; BAF, B-allele frequency. SNPs, single nucleotide polymorphisms.

**Supplementary Figure S3. Re-biopsies for aneuploid embryos positive controls.** Twelve aneuploid embryos underwent re-biopsy, with 4–6 cells collected from each of three distinct trophectoderm (TE) regions (TE_1, TE_2, and TE_3), and approximately 10 cells isolated from the inner cell mass (ICM). These embryos collectively harbored 18 aneuploid CNVs. Four segmental aberrations and one whole-chromosome aberration of mitotic origin exhibited uneven distribution across TE and ICM. In contrast, two segmental and eleven whole-chromosome aberrations of meiotic origin showed consistent distribution across both TE and ICM. -, the chromosomal abnormality was not detected in the respective re-biopsy; TE, trophectoderm; ICM, inner cell mass; BPH: both parental homologs; SPH: single parental homolog; BAF, B-allele frequency; CNVs, copy-number variations.

**Supplementary Figure S4. Prenatal diagnosis results of mosaic and aneuploid embryo transfers after PGT treatment.** (**a**) An embryo was identified with 41% deletion of chromosome five (q15q35.3)(~84.72 Mb) in PGT-A and was diagnosed as 46,XN in prenatal process. (**b**) Upper: An aneuploid embryo was identified with the same CNV of dup(15)(q25.2q25.2)(~1.52 Mb) both in PGT-A and prenatal diagnosis. Lower: An aneuploid embryo was identified with the same CNV of dup(5)(p13.2p13.1)(~4 Mb) both in PGT-A and prenatal diagnosis. PGT-A, preimplantation genetic testing for aneuploidy; CNVs, copy-number variations.

**Supplementary Figure S5. Chromosome ideograms of mosaic CNVs identified in PGT treatment and prenatal diagnosis of natural pregnancies.** (**a**) Chromosome ideograms of mosaic CNVs identified in PGT treatment. (**b**) Chromosome ideograms of mosaic CNVs identified in prenatal diagnosis of natural pregnancies. Deletion and duplication are plotted on the corresponding chromosomes, while monosomy and trisomy are indicated by counts at the chromosome ends. Pink circles, orange triangles, purple circles, and green triangles indicate mosaic deletion, mosaic duplication, mosaic monosomy, and mosaic trisomy, respectively. PGT, preimplantation genetic testing; CNVs, copy-number variations; mos_del, mosaic deletion; mos_dup, mosaic duplication; mos_monosomy, mosaic monosomy; mos_trisomy, mosaic trisomy.

**Supplementary Figure S6. The analysis results of euploid and aneuploid embryos.** (**a**) Some euploid embryos presented with mBAF deviating from 0.50, such as an embryo with genome-wide mBAF close to one due to possible genome-wide uniparental disomy (UPD), an embryo with genome-wide mBAF of ~0.67 due to maternal triploid, and an embryo with mBAF close to one in chromosome eight due to maternal UPD. mBAF was termed as the count of maternal allele as the numerator and the sum of maternal and paternal alleles as the denominator. (**b**) The validation results of two aneuploid embryos. The two aneuploid embryos were identified with chromosomal aberrations in PGT treatment, as +11; del(22)(q11.1q12.3)(~21 Mb) and dup(16)(q24.1q24.3)(~4.2 Mb). After reanalysis and re-biopsy, they were re-identified as euploid embryos. mBAF, maternal BAF; TE, trophectoderm.

**Supplementary Figure S7. Analysis of parental and cell-division origins in aneuploid embryos. (a)** Flowchart for the analysis of aneuploid embryos. A total of 167 whole-chromosome aberrations and 72 segmental aberrations were analyzed to determine their parental origin. Balanced inheritance indicated false positives, while parental bias indicated true positives. CNVs classified as true positives were subsequently analyzed to determine whether they originated from mitotic or meiotic errors. **(b)** Distribution of parental origin between different types of chromosomal aberrations. Thirteen segmental aberrations were excluded from the analysis due to insufficient SNP information. **(c)** Distribution of mitotic versus meiotic origin between different types of chromosomal aberrations. Only aberrations identified as true positives were included in the analysis. **(d)** Distribution of parental meiotic and mitotic origins between different types of chromosomal aberrations. CNVs, copy-number variations; mono, monosomy; tri, to trisomy; del, segmental deletions; dup, segmental duplication.

**Supplementary Figure S8. Analysis of parental and cell-division origins in aneuploid-mosaic embryos. (a)** Flowchart for the analysis of aneuploid-mosaic embryos. A total of 131 aneuploid CNVs and 164 mosaic CNVs were analyzed to determine their parental origin. Balanced inheritance indicated false positives, while parental bias indicated true positives. CNVs classified as true positives were subsequently analyzed to determine whether they originated from mitotic or meiotic errors. **(b)** Distribution of parental origin between different types of chromosomal aberrations in aneuploid CNVs. Twenty-six segmental aberrations were excluded from the analysis due to insufficient SNP information. **(c)** Distribution of mitotic versus meiotic origin between different types of chromosomal aberrations in aneuploid CNVs. Only aberrations identified as true positives were included in the analysis. **(d)** Distribution of parental meiotic and mitotic origins between different types of chromosomal aberrations in aneuploid CNVs. **(e)** Distribution of parental origin between different types of chromosomal aberrations in mosaic CNVs. Seven segmental aberrations were excluded from the analysis due to insufficient SNP information. **(f)** Distribution of mitotic versus meiotic origin between different types of chromosomal aberrations in mosaic CNVs. Only aberrations identified as true positives were included in the analysis. **(g)** Distribution of parental meiotic and mitotic origins between different types of chromosomal aberrations in mosaic CNVs. CNVs, copy-number variations; mono, monosomy; tri, to trisomy; del, segmental deletions; dup, segmental duplication.

**Supplementary Figure S9. Analysis of parental and cell-division origins in mosaic embryos. (a)** Flowchart for the analysis of mosaic embryos. A total of 82 whole-chromosome aberrations and 163 segmental aberrations were analyzed to determine their parental origin. Balanced inheritance indicated false positives, while parental bias indicated true positives. CNVs classified as true positives were subsequently analyzed to determine whether they originated from mitotic or meiotic errors. **(b)** Distribution of different types of chromosomal aberrations. **(c)** Distribution of parental origin between different types of chromosomal aberrations. Two segmental aberrations were excluded from the analysis due to insufficient SNP information. **(d)** Distribution of mitotic versus meiotic origin between different types of chromosomal aberrations. Only aberrations identified as true positives were included in the analysis. **(e)** Distribution of parental meiotic and mitotic origins between different types of chromosomal aberrations. CNVs, copy-number variations; mono, monosomy; tri, to trisomy; del, segmental deletions; dup, segmental duplication.

**Supplementary Figure S10. Prenatal diagnosis results of mosaic embryo transfers in PGT-A+M treatment cycles.** (**a**) An embryo was identified with 50% deletion of chromosome eight in PGT-A and was diagnosed as 46,XN in prenatal process. (**b**) An embryo was identified with 33% deletion of chromosome eight (q11.1q12.1)(~12.2 Mb) in PGT-A and was diagnosed as 46,XN in prenatal process. (**c**) An embryo was identified with 32% duplication of chromosome 15(q22.31q25.1)(~13 Mb) in PGT-A and was diagnosed as 46,XN in prenatal process. PGT-A, preimplantation genetic testing for aneuploidy; PGT-M, preimplantation genetic testing for monogenic disorders.
